# New Biogeographic insight into *Bauhinia* s.l. (Leguminosae): integration from fossil records and molecular analyses

**DOI:** 10.1186/s12862-014-0181-4

**Published:** 2014-08-10

**Authors:** Hong-Hu Meng, Frédéric MB Jacques, Tao Su, Yong-Jiang Huang, Shi-Tao Zhang, Hong-Jie Ma, Zhe-Kun Zhou

**Affiliations:** 1 Key Laboratory of Tropical Forest Ecology, Xishuangbanna Tropical Botanical Garden, Chinese Academy of Sciences, Mengla 666303, China; 2 Key Laboratory for Plant Diversity and Biogeography of East Asia, Kunming Institute of Botany, Chinese Academy of Sciences, Kunming 650204, China; 3 Faculty of Land Resource Engineering, Kunming University of Science and Technology, Kunming 650093, China; 4 Zhejiang Institute of Geological Survey, Hangzhou 311203, China; 5 University of the Chinese Academy of Sciences, Beijing 100049, China

**Keywords:** Bauhinia, Pantropical intercontinental disjunction, Evolution, Biogeography, Paleocene-Eocene thermal maximum, Boreotropical flora, Long distance dispersal

## Abstract

**Background:**

Given that most species that have ever existed on earth are extinct, it stands to reason that the evolutionary history can be better understood with fossil taxa. *Bauhinia* is a typical genus of pantropical intercontinental disjunction among the Asian, African, and American continents. Geographic distribution patterns are better recognized when fossil records and molecular sequences are combined in the analyses. Here, we describe a new macrofossil species of *Bauhinia* from the Upper Miocene Xiaolongtan Formation in Wenshan County, Southeast Yunnan, China, and elucidate the biogeographic significance through the analyses of molecules and fossils.

**Results:**

Morphometric analysis demonstrates that the leaf shapes of *B. acuminata*, *B. championii*, *B. chalcophylla*, *B. purpurea*, and *B. podopetala* closely resemble the leaf shapes of the new finding fossil. Phylogenetic relationships among the *Bauhinia* species were reconstructed using maximum parsimony and Bayesian inference, which inferred that species in *Bauhinia* species are well-resolved into three main groups. Divergence times were estimated by the Bayesian Markov chain Monte Carlo (MCMC) method under a relaxed clock, and inferred that the stem diversification time of *Bauhinia* was *ca.* 62.7 Ma. The Asian lineage first diverged at *ca.* 59.8 Ma, followed by divergence of the Africa lineage starting during the late Eocene, whereas that of the neotropical lineage starting during the middle Miocene.

**Conclusions:**

Hypotheses relying on vicariance or continental history to explain pantropical disjunct distributions are dismissed because they require mostly Palaeogene and older tectonic events. We suggest that *Bauhinia* originated in the middle Paleocene in Laurasia, probably in Asia, implying a possible Tethys Seaway origin or an “Out of Tropical Asia”, and dispersal of legumes. Its present pantropical disjunction resulted from disruption of the boreotropical flora by climatic cooling after the Paleocene-Eocene Thermal Maximum (PETM). North Atlantic land bridges (NALB) seem the most plausible route for migration of *Bauhinia* from Asia to America; and additional aspects of the *Bauhinia* species distribution are explained by migration and long distance dispersal (LDD) from Eurasia to the African and American continents.

## Background

Pantropical intercontinental disjunction is an interesting biogeographic pattern in angiosperms, and is common to several tropical and subtropical genera and families [1],[2]. However, it still remains poorly understood compared with temperate disjunctions due to greater species richness, inaccessibility of study material [3], greater ocean separation, and the vast latitudinal distribution of taxa [2]. Many pantropical taxa are hypothesized to have spanned the Northern Hemisphere during the Paleocene-Eocene Thermal Maximum (PETM), because the warmer climate of the early Palaeogene allowed thermophilic taxa to extend their ranges northward [4]. The exchange of floristic elements among pantropical regions was possible through the North Atlantic land bridges (NALB) and the Bering land bridges (BLB) during the early Eocene [5]-[8]. A “boreotropical” connection across the North Atlantic during the Eocene has long been viewed as a key to understand the disjunction patterns in the Northern Hemisphere, such as the close relationships between Eastern Asian-North American plants [9], which have been extensively studied [10]. Recently, molecular phylogenetic studies combined with molecular clock inferences have allowed a more precise understanding of the process of dispersal, and have hypothesized the divergence times of pantropical distribution patterns for many plant families, such as Melastomataceae [3],[11], Malpighiaceae [8], Annonaceae [12],[13], Myristicaceae [12], Burseraceae [14], Rubiaceae [15], Simaroubaceae [16], and Sapotaceae [1]. Among the above-mentioned studies, the results suggest that modern pantropical disjunction mostly resulted from ancestral boreotropical distribution which was disrupted by late Eocene climatic cooling. That caused the distribution ranges of plant species to shrink to lower and warmer paleolatitudes for the sake of survival, followed by migrations from North to South America [17]-[20].

Explanations for wide geographic ranges of pantropical lineages in Eurasia, America, and Africa, typically invoke three main hypotheses: (1) The vicariance hypothesis developed with the acceptance of plate tectonics theory, which has been used to explain the wide distribution lineages on portions of the ancient Gondwana continent [2],[21]. As a biogeographic mechanism, it has been proposed for the relatively older family, such as Annonaceae [12]. (2) The boreotropical migration hypothesis describes the migration of some tropical lineages between the Old and the New Worlds via the NALB or BLB during the early Tertiary Period, when the climate conditions in the Northern Hemisphere could accommodate tropical vegetations [8],[22]. This hypothesis has been proposed as an explanation for the distribution of several lineages with a classical western Gondwanan disjunction pattern, such as Burseraceae [14]. (3) Long distance dispersal (LDD) has also been proposed as a process that has significantly shaped the formation of modern biotas. This hypothesis is used, especially when the divergence times of lineages are far too young, to be explained by vicariance via tectonic plate movements [8],[21],[23]. Despite the existence of this conceptual framework, there are still relatively few well-resolved biogeographic studies on pantropical clades.

**Figure 1:**
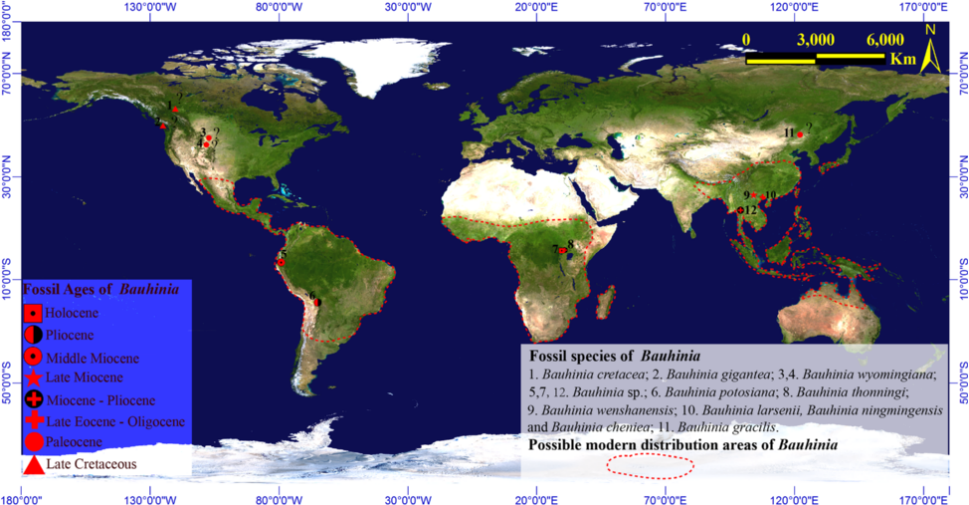
**Map showing the locations from which*Bauhinia*fossils were recovered and the modern distribution of*Bauhinia*.“?” indicates questionable fossil records.** The drawing of map is completed by the authors, and map background is courtesy of National Administration of Surveying, Mapping and Geoinformation.

*Bauhinia* L., is one of the largest genera in subfamily Caesalpinioideae (Leguminosae), comprising approximately 300 morphologically variable species of trees, shrubs, and lianas [24],[25]. It is distributed in tropical to subtropical and warm-temperate Asia, and tropical regions of Africa and the America (Figure 1). Recent studies have been revealed that the Caesalpinioideae are an early offshoot of the Leguminosae [24]-[31]. Recently, *Bauhinia* leaves were found in the Upper Miocene deposits from Wenshan, Southeast Yunnan, China. We used morphometrics to investigate the leaf shapes of *Bauhinia* and to compare the leaf shapes of extant and fossil species. Based on this new fossil record, we combined both molecular and fossil data to investigate the biogeographic history of *Bauhinia*.

Several molecular studies have focused on the phylogenetic relationships [25],[26],[32] and diversification of the legumes [33],[34]. Setting the stem node of legumes hypothetically at 60 Ma, the estimated age of the Leguminosae crown node is at 59 Ma [34]. However, the biogeographic history of *Bauhinia* is still not well resolved. A biogeographic history of *Bauhinia* should consider well-documented fossils and take into consideration dispersal in different continents. Information from pantropical flora is relatively limited, and divergence times are still unclear without fossil evidence. Well-dated, properly identified fossils and reliable phylogenetic reconstructions are crucial to reveal geographic distribution patterns. Fossils can provide direct evidence on origin time and migration or dispersal pathways to some extent, which are of special interest in biogeographic discussions when combined with molecular analyses.

In this study, we describe a new fossil species of *Bauhinia*, and use morphometrics to compare it with other species. Then, we combine fossil records and molecular analyses to discuss the timing of lineage diversifications and the biogeographic history of *Bauhinia*.

## Methods

### Geological setting

**Figure 2:**
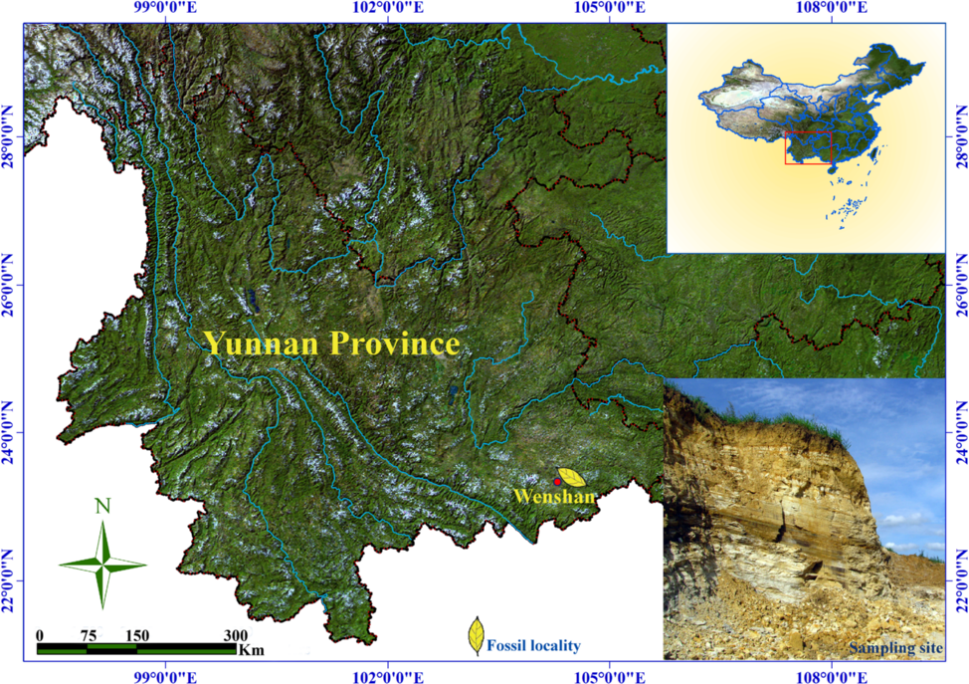
**Map showing the location of fossil locality in Wenshan, Southeast Yunnan, China.** The fossil site, labeled by a yellow leaf, is approximately 3–4 km southeast of Wenshan County. The drawing of map is completed by the authors, and map background is courtesy of National Administration of Surveying, Mapping and Geoinformation.

**Figure 3:**
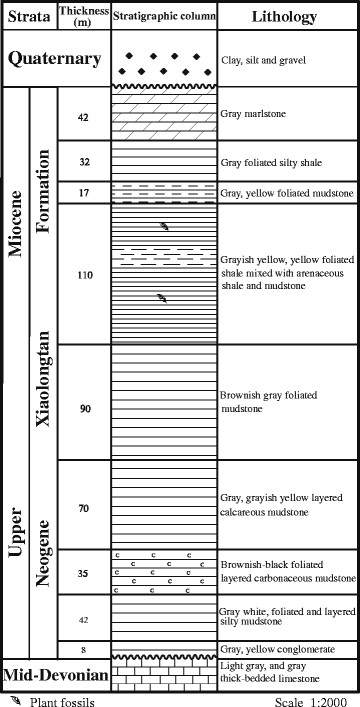
**Stratigraphy of Wenshan fossil locality.** The fossil leaves of *Bauhinia wenshanensis* were collected from a grayish-yellow, foliated shale mixed with arenaceous shale and mudstone.

Fossil leaves of *Bauhinia* were collected in Wenshan County, Southeast Yunnan, China (Figure 2). The fossil-bearing strata belong to the Xiaolongtan Formation [35]. Specimens of *Bauhinia* were preserved as compressions in marlstone and shaly siltstone from several layers of the same strata (Figure 3). The Xiaolongtan Formation is widely distributed in Central and Southeast Yunnan. Its age is assigned to the Upper Miocene Epoch according to stratigraphic correlation, palynological study [36], mammal fossils [37], and plant macrofossils [38],[39].

### *Bauhinia* fossil records

*Bauhinia* fossils have been widely reported from the late Cretaceous period to the Quaternary period [40]-[52], e.g., in Asia, Africa, North America (Table 1; Figure 1), and some wood fossils from India [53]. In Europe the only *Bauhinia* fossil record is *B. olympica* from Greece [54]. Early reports of *Bauhinia* fossils include the Cretaceous *Bauhinia cretacea* and *B. gigantea* from Canada and *B. wyomingiana* from the Cretaceous stratum of the USA [40],[42],[46], however, these *Bauhinia* records are not credible and their identification is doubtful, because they were not carefully or completely documented, or the samples only consisted of leaf fragments. We did not use *B. gracilis* as a calibration point of the root node, although it seems the oldest-known fossil records [50]. Because the holotype is a leaf with glandular teeth (Personal opinion, it may belong to Trochodendroides), rather than being an entire-margined leaf of *Bauhinia*. The original author apparently was confused by some insect damage to the leaf apex, giving a bilobed appearance vaguely similar to undamaged leaves of *Bauhinia*. But we can set the stem node of legumes hypothetically at 60 Ma to calibrate the phylogeny by following previous molecular research [34]. Extinct *Bauhinia* with confident identification includes the well-preserved *B. larsenii*, *B. ningmingensis* and *B. cheniae* from the late Eocene or early Oligocene of Guangxi in China, which has clear leaves or connect to a branch and a pod [44],[51]; and our new fossil, which has a clear leaf architecture (Table 1).

**Table 1:** **Previous published** ***Bauhinia*** **fossils and the** ***Bauhinia*** **fossils utilized in the present study**

**Fossil of** ***Bauhinia***	**Age (Ma)**	**Locality/Coordinates**	**Comments**	**Refs**
*Bauhinia gigantea*	70.6-89.3	Canada/W125°00′00",N49°45'00"	No photograph, without confidence	[40]
*Bauhinia wyomingiana*	56.8-60.2	U. S. A/W108°06'52", N42°16'44"	Leaf fragments, without confidence	[49]
*Bauhinia wyomingiana*	55.8-60.5	U. S. A/W107°10'00", N44°55'02"	Leaf fragments, without confidence	[46]
*Bauhinia cretacea*	93.5-99.6	Canada/W120°07'57", N56°13'02"	No photograph, without confidence	[41]
*Bauhinia* sp.	0-1.8	Congo/E29°49'23", N1°12'48"	Leaf, confidence in identification	[48]
*Bauhinia thonningi*	1.8-23	Uganda/E31°00'00", N1°30'00"	Leaf, confidence in identification	[45]
*Bauhinia potosiana*	1.8-5.3	Bolivia/E65°00'00", N19°00'00"	Leaf, confidence in identification	[42],[43]
*Bauhinia* sp.	11.6-15.9	Ecuador/W79°06'00", S3°23'56"	Leaf, confidence in identification	[47]
*Bauhinia larsenii*	28.4-37.2	China/E107°02'14", N22°07'41"	Branch, confidence in identification	[44]
*Bauhinia gracilis*	65.5-61.1	China/E130°25'28", N48°53'04"	Leaf fragments, without confidence	[50]
*Bauhinia* sp.	15.97-11.6	Thailand/Mae Sot, Changwat Tak	Leaf, confidence in identification	[52]
*Bauhinia ningmingensis* and *B. cheniae*	28.4-37.2	China/E107°02'14", N22°07'41"	Leaf and Branch, confidence in identification	[51]
*Bauhinia wenshanensis*	5.3-11.6	China/E104°17'19", N23°20'50"	Leaf, confidence in identification	Present study

### Specimen preparation and geometric morphometric analyses

The gross morphology of new finding fossils was compared with leaves of all the extant species of *Bauhinia* represented in Chinese National Herbarium, Institute of Botany, CAS (PE); Herbarium, Kunming Institute of Botany, CAS (KUN); and Herbarium of Xishuangbanna Tropical Botanical Garden, CAS (HITBC). We also consulted the relevant literatures about the description of *Bauhinia* leaves, such as *Flora of China*[55] and *Flora Malesiana*[56].

Images of fossil specimens were captured using a Nikon D500 Digital SLR Camera. Several images of extant species were provided by Panasonic DMC-FZ30 in herbaria. The figured specimens, and associated pictures, are deposited in paleoecology group, Xishuangbanna Tropical Botanical Garden, CAS.

Morphometric approaches described the shapes of the specimens quantitatively. We apply geometric morphometrics to investigate foliage-shape in *Bauhinia* as employed in Menispermaceae endocarps [57]. Shapes were measured for 538 extant specimens (Specimen images are from the Chinese Virtual Herbarium (CVH) at http://www.cvh.org.cn/cms/, and the Muséum national d”Histoire naturelle (P, PC), at http://www.mnhn.fr/le-museum/) (Additional file 1: Table S1), representing 81 species in East-South Asian, American and African continents, and several fossil specimens. The leaf shapes were digitized using tpsDig [58]. The contour of each leaf was divided by four lines along the leaf edge. The first of these was drawn from the pulvinus to the right lobe apex, the second from the right lobe apex to the midvein end point, the third from the midvein end point to the left lobe apex, and the fourth from the left lobe apex to the pulvinus. The pulvinus to the left, right lobe apex was described with 15 equidistant points, and the lobe apex to the midvein point with five equidistant points. All points, except end points, were analyzed as semilandmarks; there were a total of four landmarks and 36 semilandmarks. All landmarks were of type I [59]. Slider vectors were defined as the chord between the previous and next points on the curve using tpsUtil [58]. Consensus shapes were combined and partial warps and partial warp scores were calculated using tpsRelw [60]. Then, we used PAST [61] to perform principal component analysis (PCA) and cluster analysis (CA) on the partial warp scores, to explore the distribution of species in the shape space.

### Phylogeny and divergence times

The chloroplast gene tRNA-Leu (*trn*L) and the *trn*L-*trn*F intergenic spacer of 35 *Bauhinia* species and two outgroups (i.e., *Cercis chinensis* and *C. canadensis*) were downloaded from GenBank (Additional file 2: Table S2). The sequences were edited using SeqMan (Lasergene, DNASTAR Inc., Madison, Wisconsin, USA). Multiple sequence alignment was carried out in Clustal X 1.81 [62], and checked visually, refined and adjusted manually.

The combined data set was analyzed using parsimony in PAUP* 4.0b10 [63] with heuristic searching. Starting trees were obtained via stepwise addition, tree bisection reconnection branch swapping, steepest descent, and with the MulTrees and Collapse options in effect, as well as no upper limit for the number of trees held in memory. Support values for all nodes (on a 50% majority rule bootstrap tree) were calculated with the same settings as above for 1,000 replicates; 10 searches with random taxon additions were conducted for each replicate, and the strict consensus tree of all the shortest trees was saved. Bayesian inference relied on MrBayes 3.1.2 [64] and the GTR + I + G model as suggested by Modeltest 3.7 [65]. We used the default of one cold and three heated Markov chain Monte Carlo chains (MCMC), starting from random initial trees, and chains were run for 6,000,000 generations, with sampling every 200th. The default options in MrBayes were used for chain heating and mixing. We discarded a burn-in of the first 2,000,000 generations and used 20,000 trees from the posterior distribution to obtain a majority rule consensus tree.

Divergence times were estimated by BEAST 1.5.4 [66]. The BEAUti interface was used to create an input file for BEAST, in which a general time reversible (GTR) nucleotide substitution model with gamma + invariant sites was applied; an uncorrelated lognormal model was used to describe the relaxed clock. Ten million generations of the MCMC chains were run, with sampling every 1,000th. After discarding the first 1,000 trees as burn in, the samples were summarized in the maximum clade credibility tree using TreeAnnotator 1.4.8 [66], with the posterior probability limit set to 0.5, and summarizing mean node heights. Final trees were evaluated and edited in FigTree 1.3.1 [67]. Statistical support for the clades was determined by assessing Bayesian posterior probabilities. Substitution rates and the 95% highest posterior densities (HPDs) were determined with Tracer in combined runs. Divergence times are given as the mean and the 95% HPDs in millions of years, and the 95% HPDs intervals define the precision of estimation. We used the fossils to calibrate molecular dating, the stem node of legumes hypothetically at 60 Ma in molecular dating as previous molecular research [34]. The new, well-preserved, late Miocene fossil *B. wenshanensis* is hypothetically dated at *ca.*11.6 Ma according to the International Stratigraphic Chart 2010 (www.stratigraphy.org), so we used this new fossil to calibrate the crown age for the results of the morphometric analyses and the phylogenetic relationships on the basis of the ITS set (Additional file 3: Table S3).

## Results

### Systematics

*Family*: Leguminosae Juss. (or Fabaceae Lindl.)

*Subfamily*: Caesalpinioideae DC.

*Tribe*: Cercideae Bronn

*Subtribe*: Bauhiniinae (Benth.) Walp.

*Genus*: *Bauhinia* L.

*Species*: *Bauhinia wenshanensis* H.H. Meng et Z.K. Zhou sp. nov.

Twenty-four fossil species of *Bauhinia* are described as follows. All the voucher specimens were collected from the same locality and stratigraphy, and they are deposited at the same locality.

**Figure 4:**
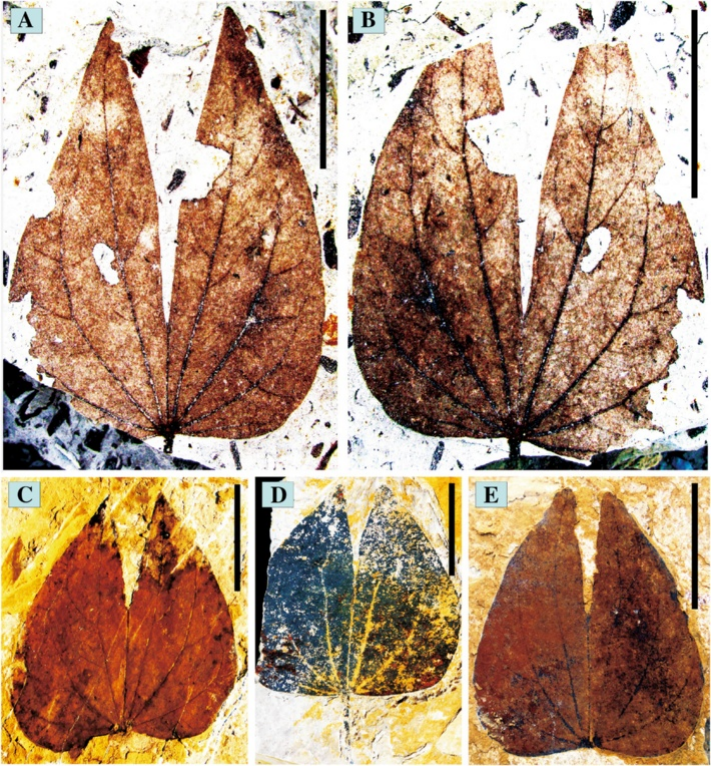
**Bauhinia wenshanensis sp. nov. from the late Miocene of Wenshan, Southeast Yunnan, China.** The leaf gross morphology of several species, (Figure 4, **A-E**). Scale bars = 2 cm.

*Holotype*: DMS0019, Figure 4A (designated here).

*Isotype*: DMS0020, Figure 4B (designated here).

*Paratypes*: DMS0008, 0021, 0022 (Figure 4C, D, E); DMS0001-0007, DMS0009-0018, DMS0023-0024 (designated here).

*Repository*: Paleoecology Research Group, Xishuangbanna Tropical Botanical Garden, CAS.

*Type locality*: Dashidong Town, Wenshan County, Southeast Yunnan, China. (23°20′50″N, 104°17′19″E, alt. 1277 m).

*Stratigraphy*: Xiaolongtan Formation, Upper Miocene Epoch.

*Etymology*: The specific epithet *wenshanensis* is given in reference to the locality Wenshan, where the fossils were collected.

*Diagnosis*: Adult leaf, petiolate, pulvinate, and simple. Lamina bilobed, apically cordate and symmetrical, apex acuminate, base rounded or ovate. Venation palmate; primary veins 7–9, midvein ending with a free, small projection beyond lamina; secondary venation camptodromous; major secondary veins terminating at the leaf edge.

*Description*: All leaf fossils are simple, petiolate, and bilobed. The basal portion is slightly ovate, rounded, or cordate. The leaf apex is obtuse, approximately 6–7 cm long and approximately 3–4 cm wide. The two lobes of the lamina share the same pulvinus. The two lobes are attached along the midvein for about 3/4 of the lamina length. Lobes are acutely pointed. The base is rounded to shallowly cordate. The petiole is 2–4 cm long, and thickened at the base. The venation is palmate with 7–9 primary veins in the leaflet. The midvein ends with a free, small apical mucro. Lateral veins are frequently branched. Secondary veins diverge at approximately 45° on the proximal side, fused with other secondary veins or the branches of primary veins to the leaf margin, or arcs between the primary veins. Higher order veins are not visible.

### Comparative morphology

**Figure 5:**
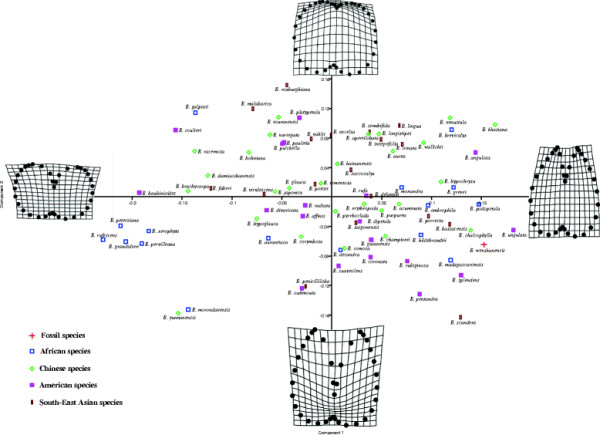
**Principal component analysis (PCA) showing the distribution of specimens according to the first and second principal components.** Graphs along axes are thin-plate splines of deformation observed along these axes. The biplot shows the contributions of the different descriptors.

**Figure 6:**
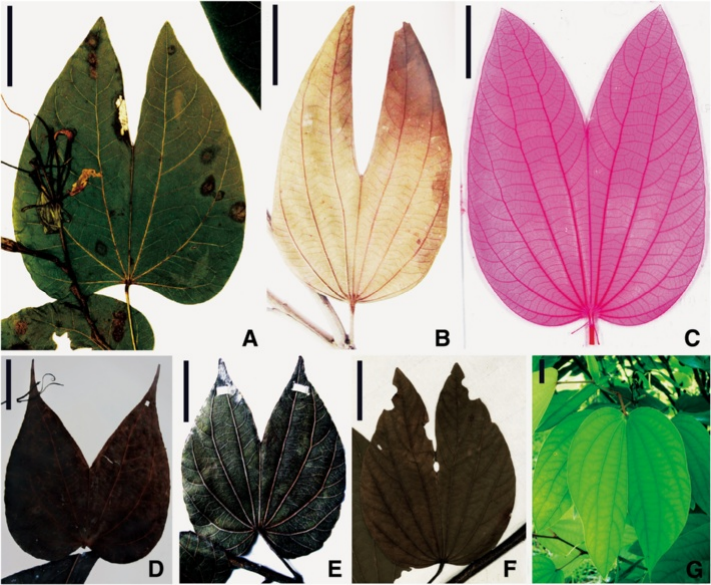
**The leaves of extant*Bauhinia*species. (A)***B. podopetala*. P00149666 (P, PC). **(B)***B. ungulata*. 01685366 (PE). **(C)** Detail of cleared lamina of *B. acuminata*. Leaf showing nearly major secondary veins and the intramarginal veins. **(D)***B. championii*. 0169748 (KUN). **(E)***B. chalcophylla*. 0169706 (KUN). **(F)***B. purpurea*. P03100612 (P, PC). **(G)** Pendent leaves of *B. acuminata.* Xishuangbanna Tropical Botanical Garden, Mengla, China, 2012. Scale bars = 2 cm.

PCA and CA among extant *Bauhinia* and the new fossil *B. wenshanensis* revealed the leaf shape variability of this genus (Figure 5 and Additional file 4: Figure S1). Species which are visibly or morphologically similar can be distinguished. However, the position of the species within the PCA and CA plots does not reflect the geographic distribution of species in *Bauhinia*. The results suggest that *B. acuminata*, *B. championii*, *B. chalcophylla*, *B. ungulata*, *B. podopetala*, *B. madagascariensis*, and *B. bassacensis* are similar to *B. wenshanensis* (Figure 5). In particular, *B. acuminata*, *B. championii*, *B. chalcophylla*, *B. purpurea,* and *B. podopetala* are the closest to *B. wenshanensis* in leaf shape (Figures 4, 5, and 6). All bear similar strongly bilobed lamina, a bifid leaflet, and a midrib ending in a mucro; especially, the leaf apex is acute. *B. acuminata*, *B. championii*, *B. purpurea,* and *B. chalcophylla*, are the most similar to the new fossil according to the PCA (Figure 6), and these species are widespread in China today. They are bilobed with a shared pulvinus, and the leaf blade parted to approximately 1/3 to 1/2 of its length.

### Phylogenetic relationships and divergence times

**Figure 7:**
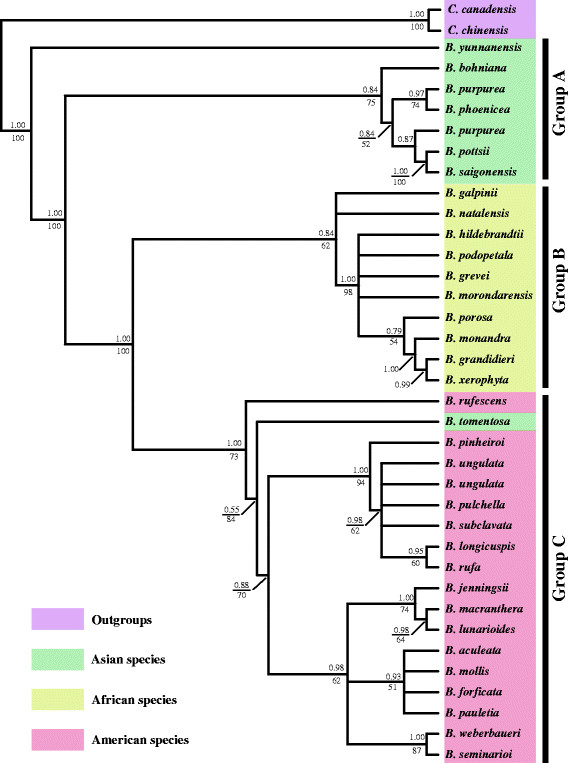
**Phylogenetic relationships among extant*Bauhinia*species based on the cpDNA tRNA-Leu (*trn*L) gene and the*trn*L-*trn*F intergenic spacer data.** The numbers above the branches are support values from Bayesian inference/bootstrap resampling.

The most parsimonious tree and the Bayesian inference tree yielded essentially identical topologies, so only the Bayesian analysis tree is illustrated here (Figure 7). The phylogram shows that *Bauhinia* species are well-resolved into three main groups. The clades with the high bootstrap values (BP) in the maximum parsimony (MP) analysis also had high posterior probabilities (PP) in the Bayesian analysis. The three main clades recognized in the phylogeny are groups A and B, which include the species in Asia and Africa respectively; and group C, which includes the species in America and *B. tomentosa,* an Asian species (Figure 7).

**Figure 8:**
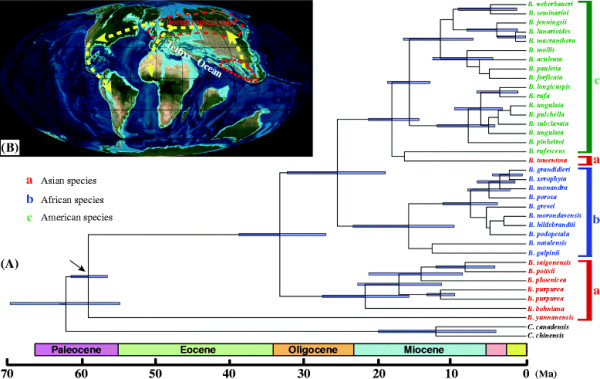
**The divergence time and possible dispersal routes. (A)** Bayesian divergence time estimates of *Bauhinia* based on the cpDNA. The blue bars on the nodes indicate 95% posterior credibility intervals. The arrow indicates the stem node of legumes hypothetically at 60 Ma to calibrate the stem age in molecular dating as previous molecular research [34]. **(B)** The possible dispersal routes of *Bauhinia*. The paleogeographic map is from Colorado Plateau Geosystems, Inc. Reconstructing the Ancient EARTH.

The Bayesian estimation of divergence times for the three major clades of *Bauhinia* is presented along with calibration points from fossil records on a chronogram (Figure 8A). The estimated initial divergence time of *Bauhinia* is *ca.* 62.7 Ma (Paleocene), and the divergence time of the Asian species *B. yunnanensis* was *ca.* 59.8 Ma (Paleocene). The subsequent divergence between the Asian and African-American species groups is estimated at *ca.* 34.3 Ma (late Eocene), and the diversification times of subclades of the Asian, African, and American species group were *ca.* 21.8 Ma, *ca.* 15.3 Ma, and *ca.* 18.7 Ma, respectively.

## Discussion

### Fossil leaves of *Bauhinia*

Palmately veined leaves that are divided into two halves, either fused or free, each half with independent nyctinastic movement but borne on a single apical common joint, are unique features of the Leguminosae family [24],[30],[68]. There are 12 genera in the tribe Cercideae with unifoliolate or bifoliolate leaves, and several of the unifoliolate taxa (e.g., *Piliostigma*, *Tylosema*, *Lysiphyllum*, and many *Phanera* species) with bilobed leaves [69]. However, the secondary veins of these species are entirely different among them. In China, *Phanera championii* is the homotypic synonym of *Bauhinia championii*, the present fossil was carefully compared, and these were found to be very similar, with the exception of the secondary veins (Figure 6D). The leaves have moderately developed secondary veins on the midrib and interprimary veins that are easily distinguished from *Bauhinia*[70], which are incongruent with the secondary veins of the present fossils. Therefore, the present species is thus obviously referred to as *Bauhinia*.

After comparing the fossil materials with specimens of extant species of the genus, we conclude that the fossil specimens represent a hitherto undescribed taxon of *Bauhinia*. The morphometric analysis demonstrates the uniqueness of the leaf shape of the fossils. Therefore, a new species is proposed.

### Implications of *Bauhinia* fossil history

We used *Bauhinia wenshanensis* to calibrate the crown age. *B. wenshanensis* is morphologically most similar to *B. acuminata* and *B. purpurea* according to the PCA and CA that were carried out in the morphometric approach. The phylogenetic analyses also illustrated that *B. acuminata* and *B. purpurea* are the closest genetic relatives on the basis of the ITS sequence (Additional file 3: Table S3, Additional file 5: Figure S2), so *B. wenshanensis* was used to calibrate the crown age of *Bauhinia* and constrain the age of *B. purpurea*.

The dating of evolutionary events, such as in phylogeny, biogeography, and evolution studies, has frequently relied on fossils because of their special significance [71]-[75]. The distribution area of the oldest lineage has often been regarded as the original center or diversity center of the taxon [76]. East Asia is one distribution center of some genera of pantropical floral distribution patterns, such as *Bauhinia*[77]. However, the regions where the oldest known fossils occur might not be the center of origin of the genus [78],[79]. To explore the center of origin, we need to integrate molecular data and fossil taxa in the analyses.

### Phylogenetic implications

The previous phylogeny of *Bauhinia* s.l. used 85 representative species, and indicated that *Bauhinia* was paraphyletic with the monospecific genus *Brenierea* clustered within it, but *Brenierea* is usually described as sister to *Bauhinia*[25]. Bayesian analysis well resolves and reflects a phylogeny of the extant *Bauhinia* (Figure 7). Our results revalidated the sister-group relationships and supported three major clades of *Bauhinia*, groups A, B, and C, representing the *Bauhinia* species in Asian, African, and the American continents respectively (Figure 7). Group A includs the representative species from Asia, which are all native Asian species from China, Singapore, Thailand and Australia. Group B included the species from Africa (e.g., Mauritius and Madagascar), which were unambiguously supported to be monophyletic. Group C represents species growing in the Neotropics; however, the species *B. tomentosa* is nested within group C. *B. tomentosa* is considered to have originated in tropical Asia, and is probably indigenous to India, although widely cultivated as an ornamental plant [55], and *B. tomentosa* from Hawaii was considered as cultivated species in previous research [25]. Thus, the relationship between *B. tomentosa* and the other species in group C indicates the close affinity between this Asian species and those of the American continent.

### Biogeographic implications of *Bauhinia*

Based on our calibration point including the hypothetical stem age lacking fossil evidence, plus the crown age based on our fossil, the divergence of *Bauhinia* from its closest relative lineage in the Leguminosae family occurred at *ca.* 62.7 Ma (Figure 8A). The biogeographic scenario that *Bauhinia* originated and initially diversified in Asia is tenable, and consistent with the greatest extant diversity and the highest endemism in Asia (e.g., 23 in 47 species are endemic in China according to *Flora of China*) [55]. The Asian group is more basal than the American and African groups, and *B. yunnanensis* is a relatively primitive species, which also suggests that East Asia should be the earliest diversification center.

Here, hypotheses based on vicariance or continental history to explain continental disjunct distributions are dismissed in the biogeography of *Bauhinia*, because the hypotheses mostly require Palaeogene or older tectonic events [80]. According to our results, *Bauhinia* originated in Asia and then dispersed in Africa and America. We make the hypothesis that the genus scattered circumboreally across the Northern Hemisphere, although there only fossil record in Europe, *Bauhinia olympica* (No photograph and just a record; maybe questioned), is from Greece [53], and there are extant plants in North America but without valid *Bauhinia* fossil records. Then, it entered the North American floristic area through the BLB or the NALB; these land connections and widespread equable climates allowed a relatively homogeneous boreal flora to distribute through a large part of the Northern Hemisphere during the Eocene Epoch [81]-[86].

Oxygen isotope records suggest that very warm climates occurred globally in the late Paleocene to the early Eocene, but cooling proceeded during the mid- to late Eocene with small fluctuations [84]. The drastic cooling events after the PETM made the boreotropical floral elements move to lower latitudes, leading to segregation of the ancestral lineages of modern tropical plants between the North American and Eurasian continents [17],[18],[20],[87]. *Bauhinia* might have followed the same path and retreated from high to lower latitudes and warmer regions to survive. The absence of extant *Bauhinia* in Europe does not immediately rule out the NALB as a possible migration pathway. The BLB is situated at higher latitudes than the NALB, which restricted such a tropical or subtropical plant to migrate (e.g., ecological factors, light and temperature). So the NALB might have been the only migration route between Eurasia and America available at any point during the Tertiary for *Bauhinia*, and should be considered as a migration route for direct migration between southwest Eurasia and North America. The ‘Madrean-Tethyan’ route, wherein a string of volcanic islands at the latitude of the modern Azores allowed migration by ‘island-hopping’ between the two continents between 25 and 38 Ma [22],[88],[89] is also possible. Many tropical taxa have undergone dispersal through the NALB, Malpighiaceae, the “American colonist” scenario had required at least six dispersal events across the Atlantic [8]; *Ampelopsis* had two independent migrations into Eurasia that are inferred to have occurred in the early Miocene via the NALB [5]. The discovery of more fossil records in Europe would support the NALB route robustly. Here, we suggest that a scenario in which *Bauhinia* first migrated from Asia to Europe, and then to the America through the NALB, seems the most plausible (Figure 8B).

Additionally, we suggest that the African lineage migrated into Africa via Eurasian continent, particularly from Eurasia to Africa (Figure 8B). The split between the Asian and African-American lineages during the late Eocene to early Oligocene generally coincides with periods of cooling across northern latitudes [90],[91]. This period may also correspond to a time when ancestral acridocarpoids (Malpighiaceae) followed equable climates southward [8]. Additionally, dispersal from Eurasia into Africa seems likely in view of the ancient connections between Africa and Eurasia. Five episodes of major regional change in palaeogeographic and tectonic settings are recognized: late Eocene (37–34 Ma), early late Oligocene (30–27 Ma), latest early to earliest middle Miocene (17–15 Ma), early late Miocene (9–8 Ma), and late early to early middle Pliocene (4–3 Ma) [92]. Most interestingly, this early middle Miocene Africa-Eurasia convergence could explain the *Bauhinia* migration from Eurasia to Africa and then diversification (diversification time of African subclade is at *ca*. 15.3 Ma), that inferred from our analyses.

The palaeogene fossil records of *Bauhinia* are not reliable, and the scenarios that reliable macrofossils of *Bauhinia* made their debut in the Eocene-Oligocene floras from mid-low latitudes and appeared to lack in the boreotropical floras seem more reasonable; however, we suggest that *Bauhinia* may have been in the coeval floras at high latitudes (i.e., palaeogene floras in North America and Europe) but not in the right place or time for fossils to be preserved. In addition, the evidence from integrated fossil and molecular evidence supports a tropical Tethys Seaway origin and spread or an “Out of Tropical Asia” dispersal of the Cercideae and the Leguminosae [25],[51]. Under this scenario, we suggest that *Bauhinia* originated in Asia during the Paleocene and then spread out to other regions, although they were in Asia by the early Oligocene based on direct fossil evidence [44],[51]. Such a scenario is consistent with the hypothesis of the Indomalayan region being a refugium for the boreotropical flora during the global cooling [7],[93]. Lowland tropical forests in this region have experienced relatively climatic and ecological stability since the late Cretaceous in contrast to their American and African counterparts [94], although the uplift of Himalayas and the monsoonal intensification have impacted these regions. Moreover, land connections between Southeast Asia and more northerly regions of the Northern Hemisphere were presented during much of the Tertiary and could have provided migration routes between these areas [95],[96]. The middle Miocene and Holocene fossils in Africa confirm that *Bauhinia* has been found in Africa since the middle Miocene and is consistent with a migration from Eurasia into Africa. Thus, our data suggest that *Bauhinia* migrated into Africa from Eurasia and then subsequently into Madagascar.

Nevertheless, the possibility of long distance dispersal (LDD) events from Eurasia to the African and American continents cannot be ruled out. LDD has been viewed as a dominant mechanism for the distribution of many relatively younger tropical plant lineages, such as taxa in Chrysophylloideae [1], Simaroubaceae [16], and Melastomataceae [11]. Some studies have already indicated that LDD events are caused by random incidents [11],[97]-[99], and the dispersal mechanism of the plant itself is sometimes irrelevant for LDD [100]. However, LDD has also been invoked as an explanation for the pantropical disjunction of *Bauhinia*. We suggest that birds are major agents of *Bauhinia* plant dispersal, because the birds, with long-distance flight capabilities, are considerably important for leguminous species [101], and the dispersal mechanism of legumes was inferred [102].

## Conclusions

We describe a new species of *Bauhinia* from the late Miocene in Wenshan, Southeast Yunnan, China. Analyses integrating hypothesized age of the stem group, plus fossil and molecular data, suggest that the present pantropical distribution pattern was already established by the middle Paleocene in Laurasia, and possibly originated from Asia. The disjunct distribution of *Bauhinia* in America, Africa, and Asia is the result of the southward movement of the boreotropical flora in response to the climatic cooling during the late Eocene to the early Oligocene. After originating in Asia, *Bauhinia* reached the whole of Eurasia and then migrated to the American continent via the NALB, although there are no validly determined *Bauhinia* fossils in the North American record. And *Bauhinia* migrated from Eurasia to Africa when the two land masses were connected during the Cenozoic Era. LDD can also explain some of the patterns which were observed. The findings illustrate the power of incorporating fossil records and modern plant distribution areas, together with assumptions about stem group age, to better understand the geographic patterns and the possible dispersal routes of plant species.

## Competing interests

The authors declare that they have no competing interests.

## Authors’ contributions

ZKZ conceived and conducted the study. HHM performed the data analyses, evolutionary interpretations, and wrote the manuscript. FMBJ performed data analyses. TS photographed the specimens and revised the manuscript. HHM, FMBJ, TS, YJH, STZ and HJM collected the type specimens. All authors read and approved the final manuscript.

## Additional files

## Supplementary Material

Additional file 1: Table S1 List of studied specimens. The numbers in the table after a genus name refer to the number of recognized species in the genus. Click here for file

Additional file 2: Table S2 GenBank accession numbers and their references for sources of cpDNA tRNA-Leu (*trn*L) gene and *trn*L-*trn*F intergenic spacer data of *Bauhinia* and the outgroups. Click here for file

Additional file 3: Table S3 GenBank accession numbers and their references for sources of ITS (rRNA internal transcribed spacers) data of *Bauhinia* and the outgroups. Click here for file

Additional file 4: Figure S1 The cluster analysis (CA) dendrogram of the *Bauhinia* leaf shape. Click here for file

Additional file 5: Figure S2 The phylogenetic relationships among extant *Bauhinia* species based on the rDNA ITS. The numbers above the branches are support values from Bayesian inference/bootstrap resampling. Click here for file
