# Supplementary material for: New Biogeographic insight into Bauhinia s.l. (Leguminosae): integration from fossil records and molecular analyses
Source: BMC Evol Biol. 2014 Aug 10;14:181. doi: 10.1186/s12862-014-0181-4 (PMC4360257; doi:10.1186/s12862-014-0181-4)
Supplement: Additional file 1: Table S1 — List of studied specimens. The numbers in the table after a genus name refer to the number of recognized species in the genus. [file s12862-014-0181-4-S1.doc]

**Table S1** List of studied specimens. The numbers in table after a genus name refer to the number of recognized species in the genus.

| Locality | Specimens of *Bauhinia* and the number of specimens |
| --- | --- |
| **China** | *B. acuminata*. KUN 0169191, 0169185, 0125369, 0169182, 0169186, 0169183; *B. tomentosa*.KUN 0125179, HITBC 097771, 097767, 097683, 105362, 017502; *B. erythropoda*. KUN 0125173, 0169816, 0169813, 0169815, 0169814; *B. comosa*. KUN 0125121, 0125122, 0169773, 0169774, 0169778; *B. yunnanensis*. KUN 0157575, 0125364, 0125311, 0125353, 0125333, 0125349, *B. chalcophylla*. KUN 0169706, PE 00020914, 00323866, 00327886, HITBC 017342; *B. championii*. KUN 0169748, 0169733, 0169751; *B. japonica*. KUN 0125093-0125097; *B. purpurea*. P,PC P03100612, KUN 0125148, 0125149, 0125151, 0125154; *B. glauca*. KUN 0125012, 0125010, 0125018, 0169922, 0169903; *B. hypoglauca*. IBK 00068645, IBSC 0161712; *B. pyrrhoclada*. PE 00324321, 00324463, 00324464, 00324465, 00324466, 00324467; *B. corymbosa*. PE 00323870, 00323871, 00323873, 00323874, 00323875; *B. apertilobata*. KUN 0169196, 0169213, 0169197, 0169214, 0169216; *B. longistipes*. KUN 0125119, 0125118; *B. hainanensis*. KUN 0125092, 0125088, 0125089; *B. aurea*. KUN 0169201, 0169221, 0169205, 0169202, 0169198, 0169206; *B. wallichii*. IBSC 0161872, 0161871; *B. khasiana*. KUN 0125099, 0125105, IBK 00068661; *B. venustula*. KUN 0125297, IBSC 0161868; *B. hypochrysa*. PE 00324080, IBSC 0161710; *B. brachycarpa*. KUN 0169252, 0169303, 0169291, 0169293, 0169323, 0169417, 0169320; *B. damiaoshanensis*. HITBC 017416-017419; *B. racemosa*. KUN 0125158, 0125160, 0125165, 0125171, 0125169, 0125162, 0125168; *B. bohniana*. KUN 0169247, 0169244, 0169242, 0169249, 0169241; *B. variegate*. KUN 0125257, 0125243, 0125242, 0125246, 0125256; *B. touranensis*. KUN 0125181, 0125233, 0125197, 0125221, 0125211, 0125180. |
| **East-South Asia** | *B. porrecta*. P,PC P02987930, P02999580, P03100591-P03100595; *B. delavayi*. P,PC P02999618, P03100115, P03100119, P03100122, P03100120; *B. saigonensis*. P,PC P00798505- P00798512; *B. saccocalyx*. P,PC P02744853- P02744858; *B. pottsii*. P,PC P02999624, P03449414, P03449415, P03449419, P03449421, P03449422; *B. viridescens*. P,PC P02746124, P00087785, P02746140, P02746143, P02746147, P02746505, P03650945; *B. penicilliloba*. P,PC P00798530, P00798531, P02744723, P02744724, P02744726, P02744727, P02744732; *B. excelsa*. P,PC P00798559, P03449995, P03449996; *B. semibifida*. P,PC P02744752 -P02744754, P03449691, P03449693, P03449698, P03449706, P03449709; *B. vahlii*. P,PC P02746254, P02746258, P02746260, P02746262, P02746263, P02746270, P02746272; *B. integrifolia*. P,PC P030137, P030139, P00087785, P03449532, P03449576; *B. ornate*. P,PC P00752516, P00798521, P00798523, P02744705, P02744710, P02744713; *B. lingua*. P,PC P03449463, P03449464; *B. bassacensis*. P,PC P00798470, P00798473, P00798474, P03100715, P03100723; *B. scandens*. P,PC P02999574, P02999573, P02744778; *B. faberi*. P,PC P03100804, P03100807, P03100815, P03100817, P03100833; *B. roxburghiana*. P,PC P02744833, P02744836, P02744841; *B. malabarica*. P,PC P00293965, P00761790, P02744664, P03449451, P03449447, P03449404. |
| **Africa** | *B. aurantiaca*. P,PC P00129943-P00129948, P02965538; *B. brevicalyx*. P,PC P00090381, P00131610, P00131616, P00701733, P03080258; *B. decandra*. P,PC P00090802-P00090805, P00139281, P00724697, P00090822; *B. galpinii*. P,PC P02746609, P03500557, P03500560, P03500563, P03500568, P03500569; *B. grandidieri*. P,PC P00129901, P00129921, P00129857; *B. grevei*. P,PC P00131957, P00137052, P00137061, P00137085, P00137100; *B. hildebrandtii*. P,PC P023709, P00149459, P00149466, P00149506, P00334013; *B. madagascariensis*. P,PC P03080259, P02987920, P00724534, P00149535, P030008; *B. monandra*. P,PC P00149616, P03100663, P03449390, P00149590; *B. morondavensis*. P,PC P00090366, P00090368, P00090378, P00118389, P00724729; *B. ombrophila*. P,PC P00090353, P00090358, P00090360, P00751228; *B. pervilleana*. P,PC P00131703, P00131707, P00149577, P00149578, P00149580; *B. petersiana*. P,PC P02869745, P03500596, P03500615, P03500619, P03500622, P03500624; *B. podopetala*. P,PC P00131713, P00149645, P00149653, P00149654, P00149666, P00149670; *B. rufescens*. P,PC P03481257, P03481270, P03500707, P03500936, P03500947; B. xerophyta. P,PC P023580, P00090388, P00090391. |
| **Americanum** | *B. ungulate*. PE 01685366, P,PC 01685366, P03449900, P03449914, P03449915, P03449951; *B. guianensis*. P03450084, P03450086, P03450093, P03450112, P03450123, P03450217; *B. dipetala*. P00798567, P02746395, P02746401, P02746404, P02746405, P02746406; *B. rufa*. P03449322, P03449327, P03449349, P03449355; *B. divaricata*. P02746346, P02746348, P02746349, P02746365, P02746373, P03333901, P03490022; *B. affinis*. P00758979- P00758981, P00758983, P00758984, P00758986; *B. radiate*. P02999552- P02999552, P03449713, P03449715-P03449717; *B. outimouta*. P03450234- P03450237, P03450270, P03450266; *B. suaveolens*. P00679203, P00756095, P03449089-P03449091; *B. angulosa*. P00758976- P00758978, P02746703-P02746706; *B. coronata*. P02746814, P02746815, P02746817-P02746821; *B. rubiginosa*. P03449738-P03449747; *B. splendens*. P03449058, P03449059, P03449062, P03449063, P03449065-P03449067; *B. pentandra*. P03450306, P03450307, P03450309, P03450310, P03450312 -P03450318; *B. bauhinioides*. P00758972, P02746693, P02746696, P02746698; *B. coulteri*. P02746803- P02746805, P02746807, P02746810-P02746813; *B. platypetala*. P00798596, P00798597, P03449774 -P03449779; *B. pauletia*. P00798621, P03450274, P03450275, P03450277, P03450280, P03450281; B. pulchella. P00798601, P00798602, P03449780, P03449783-P03449785. |
