# Supplementary material for: New Biogeographic insight into Bauhinia s.l. (Leguminosae): integration from fossil records and molecular analyses
Source: BMC Evol Biol. 2014 Aug 10;14:181. doi: 10.1186/s12862-014-0181-4 (PMC4360257; doi:10.1186/s12862-014-0181-4)
Supplement: Additional file 2: Table S2 — GenBank accession numbers and their references for sources of cpDNA tRNA-Leu (trnL) gene and trnL-trnF intergenic spacer data of Bauhinia and the outgroups. [file s12862-014-0181-4-S2.doc]

Table S2 GenBank accession numbers and their references for source of cpDNA tRNA-Leu (*trn*L) gene and *trn*L-*trn*F intergenic spacer data of *Bauhinia* and the outgroups.

| Section | Taxon | Locality | NCBI no. | Reference |
| --- | --- | --- | --- | --- |
| *Bauhinia* | *B. yunnanensis* | Fairchild Tropical Garden | FJ801084 | Sinou *et al*. (2009) |
|  | *B. bohniana* | Royal Botanic Garden, Melbourne | FJ801052 | Sinou *et al*. (2009) |
|  | *B. purpurea* | Singapore Botanical Garden | FJ801075 | Sinou *et al*. (2009) |
|  | *B. purpurea* | Australia | FJ801069 | Sinou *et al*. (2009) |
|  | *B. pottsii* | Thailand | FJ801077 | Sinou *et al*. (2009) |
|  | *B. saigonensis* | Singapore Botanical Garden | FJ801114 | Sinou *et al.* (2009) |
|  | *B. phoenicea* | India | FJ801151 | Sinou *et al.* (2009) |
|  | *B. galpinii* | Mauritius | FJ801055 | Sinou *et al.* (2009) |
|  | *B. hildebrandtii* | Madagascar | FJ801060 | Sinou *et al.* (2009) |
|  | *B. podopetala* | Madagascar | FJ801129 | Sinou *et al.* (2009) |
|  | *B. porosa* | Madagascar | FJ801135 | Sinou *et al.* (2009) |
|  | *B. grevei* | Madagascar | FJ801147 | Sinou *et al.* (2009) |
|  | *B.* *monandra* | Madagascar | FJ801127 | Sinou *et al.* (2009) |
|  | *B.* *grandidieri* | Madagascar | FJ801132 | Sinou *et al.* (2009) |
|  | *B.* *morondavensis* | Madagascar | FJ801130 | Sinou *et al.* (2009) |
|  | *B. xerophyta* | Madagascar | FJ801128 | Sinou *et al.* (2009) |
|  | *B. natalensis* | Kristenbosch Botanical Garden | FJ801064 | Sinou *et al.* (2009) |
|  | *B. tomentosa* | Hawai, USA | FJ801088 | Sinou *et al.* (2009) |
|  | *B. rufescens* | Florida, USA | FJ801082 | Sinou *et al.* (2009) |
|  | *B. pinheiroi* | Brazil | FJ801139 | Sinou *et al.* (2009) |
|  | *B. ungulata* | Costa Rica | FJ801073 | Sinou *et al.* (2009) |
|  | *B. ungulata* | Mexico | FJ801110 | Sinou *et al.* (2009) |
|  | *B. pulchella* | Brazil | FJ801097 | Sinou *et al.* (2009) |
|  | *B. subclavata* | Brazil | FJ801109 | Sinou *et al.* (2009) |
|  | *B. longicuspis* | Bolivia | FJ801095 | Sinou *et al.* (2009) |
|  | *B. rufa* | Brazil | FJ801098 | Sinou *et al.* (2009) |
|  | *B. jenningsii* | Belize | FJ801062 | Sinou *et al.* (2009) |
|  | *B. macranthera* | Mexico | FJ801063 | Sinou *et al.* (2009) |
|  | *B. lunarioides* | Mexico | FJ801141 | Sinou *et al.* (2009) |
|  | *B. aculeata* | Venezuela | FJ801051 | Sinou *et al.* (2009) |
|  | *B. mollis* | Bolivia | FJ801134 | Sinou *et al.* (2009) |
|  | *B. forficata* | Paraguay | FJ801094 | Sinou *et al.* (2009) |
|  | *B. pauletia* | Nicaragua | FJ801087 | Sinou *et al.* (2009) |
|  | *B. weberbaueri* | Peru | FJ801103 | Sinou *et al.* (2009) |
|  | *B. seminarioi* | Ecuador | FJ801102 | Sinou *et al.* (2009) |
| *Cercis* | *C. canadensis* | Montreal Botanical Garden | FJ801162 | Sinou *et al.* (2009) |
|  | *C. chinensis* | US Botanical Garden | FJ801081 | Sinou *et al.* (2009) |
