# Supplementary material for: New Biogeographic insight into Bauhinia s.l. (Leguminosae): integration from fossil records and molecular analyses
Source: BMC Evol Biol. 2014 Aug 10;14:181. doi: 10.1186/s12862-014-0181-4 (PMC4360257; doi:10.1186/s12862-014-0181-4)
Supplement: Additional file 3: Table S3 — GenBank accession numbers and their references for sources of ITS (rRNA internal transcribed spacers) data of Bauhinia and the outgroups. [file s12862-014-0181-4-S3.doc]

Table S3 GenBank accession numbers and their references for source of ITS (rRNA internal transcribed spacers) data of *Bauhinia* and the outgroups.

| Section | Taxon | NCBI no. | Reference |
| --- | --- | --- | --- |
| *Bauhinia* | *B. yunnanensis* | AF286360 | Hao *et al*. (2000) |
|  | *B. bohniana* | AY258403 | Hao *et al*. (2003) |
|  | *B. purpurea* | JQ673525 | Barishi *et al*. (2012) |
|  | *B. acuminata* | AY126642 | Mak *et al*. (2008) |
|  | *B. purpurea* | AF387973 | Mak *et al*. (2008) |
|  | *B. championii* | AY258377 | Hao *et al.* (2003) |
|  | *B. gilva* | AY258401 | Hao *et al.* (2003) |
|  | *B. glabra* | AY258409 | Hao *et al.* (2003) |
|  | *B. brachycarpa* | FJ432276 | Coskun and Parks (2009) |
|  | *B. macranthera* | JN942381 | Fritsch and Cruz (2012) |
|  | *B. jenningsii* | AY258411 | Hao *et al.* (2003) |
|  | *B. pyrrhoclada* | AF256359 | Hao *et al.* (2000) |
|  | *B. hannanensis* | AY258407 | Hao *et al.* (2003) |
|  | *B. japonica* | AF256358 | Hao *et al.* (2000) |
|  | *B.* *strychniflolia* | AY258405 | Hao *et al.* (2003) |
|  | *B. ungulata* | FJ005818 | Conceicao *et al.* (2009) |
| *Cercis* | *C. canadensis* | JQ425127 | Wadl *et al.* (2012) |
|  | *C. chingii* | JQ425125 | Wadl *et al.* (2012) |
